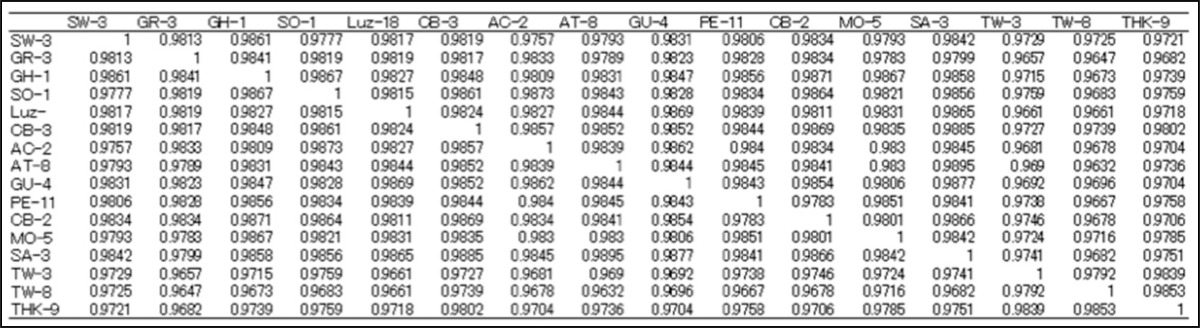# Detection of Human Polyomavirus DNA Using the Genome Profiling Method

**DOI:** 10.2174/1874357901509010029

**Published:** 2015-11-24

**Authors:** Yuka Tanaka, Rieko Hirata, Kyohei Mashita, Stuart Mclean, Hiroshi Ikegaya

**Affiliations:** Department of Forensic Medicine, Kyoto Prefectural University of Medicine Graduate School of Medical Sciences, 465 Kajii-cho Kawaramachi-Hirokoji, Kamigyo-ku, Kyoto 602-8566, Japan

**Keywords:** Genome profiling method, JC virus, BK virus, urine, temperature gradient gel electrophoresis, random PCR

## Abstract

**Background:**

In the field of forensic medicine, it is very difficult to know prior to autopsy what kind of virus has infected a body.

**Objective:**

We assessed the potential of the genome profiling (GP) method, which was developed in the field of bioengineering, to identify viruses belonging to one species.

**Method:**

Two species in the same family, JC and BK viruses, were used in this study. Using plasmid samples, we compared the findings of molecular phylogenetic analysis using conventional genome sequencing with the results of cluster analysis using the random PCR-based GP method and discussed whether the GP method can be used to determine viral species.

**Results:**

It was possible to distinguish these two different viral species. In addition to this, in our trial we could also detect the JC virus from a clinical sample.

**Conclusion:**

This method does not require special reagent sets for each viral species. Though our findings are still in the trial period, the GP method may be a simple, easy, and economical tool to detect viral species in the near future.

## 1 INTRODUCTION

**Fig. (1):**
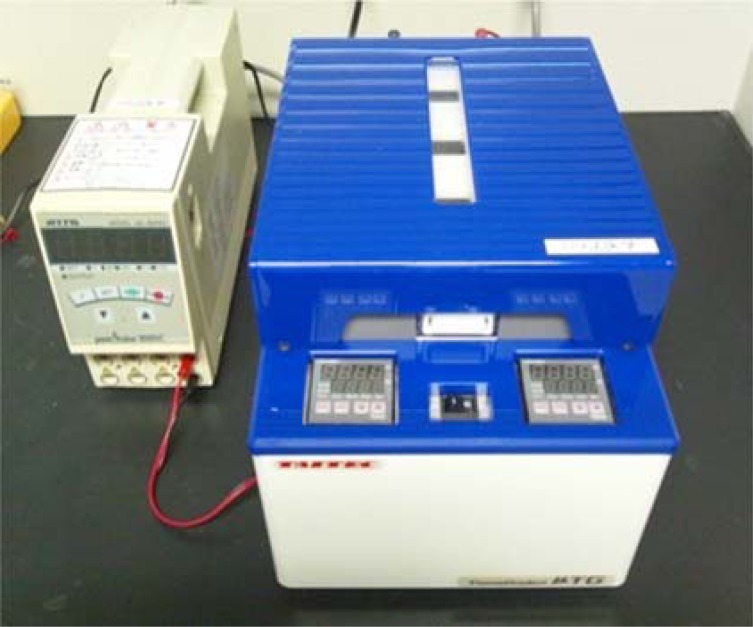
Temperature gradient gel electrophoresis apparatus (µTG; TAITEC, Saitama, Japan).

The genome profiling (GP) method, which was developed in the field of bioengineering in 2000 by Nishigaki *et al.* [1] can be used to distinguish differences between genomes using a random PCR approach and temperature gradient gel electrophoresis (Fig. **1**). In the field of virology, sequencing and phylogenetic analyses are usually employed to analyze viral genomes. However, these approaches require considerable effort, expensive equipment, and specific reagents. Conversely, the GP method is reportedly a very simple tool to analyze whole genomes and requires inexpensive equipment and versatile reagents [2].

The first step of the GP method consists of PCR using random primers, which amplifies the whole genome partially and randomly. This process is considered almost the same as random sampling from the whole genome. Therefore, analysis of the randomly generated PCR fragments represents whole genome information. In the second step of the GP method, the amplified DNA fragments are electrophoresed in a temperature gradient polyacrylamide gel. With this gel, sequence-specific temperature denaturation points are obtained. We call these points “species identification dots” (*spiddos*). By analyzing these *spiddos *with standards, similar genome samples are recognized as a single cluster. 

The GP method has been used to distinguish mammalian species [2] and human body fluid types [3, 4] in the field of forensic medicine. However, it has not been used to distinguish species of virus. In forensic medicine, we usually examine cadavers with unknown histories. Therefore, for the protection of pathologists and other people who deal with the body, it is very important to know what infectious microbes they may carry. Recently there have been outbreaks of many newly emerging infectious diseases, including the Ebola virus, SARS virus, MEARS virus, avian influenza and so on. However, it is not easy to detect those viruses without special equipment and reagents.

In the present study, we assessed the potential of the GP method to identify viral species, using the JC virus (JCPyV) and BK virus (BKPyV) as representative microbes. These viruses belong to the *polyomaviridae *family, which consists of 5200 base-pair (bp) double-stranded DNA viruses [5, 6]. We considered that it would be very easy to compare the conventional identification method with the GP method using these viruses as they have a relatively small genome and are DNA viruses, making it easy to analyze whole genomes by both methods.

JCPyV was first isolated from the brain of a patient with progressive multifocal leukoencephalopathy (PML) in 1971 by Padgett *et al.* [7]. Recently, PML has become an important disease in patients with human immunodeficiency virus infection [8]. Previous studies showed that JCPyV infection is ubiquitous in humans [9, 10]. After the initial infection, JCPyV persists in the kidney. It is detectable in the urine of 20–70% of healthy individuals, and its infection rate increases with age [10-13].

BKPyV was isolated in 1971 from the urine of a renal transplantation patient [14]. In bone marrow transplant patients, BKPyV induces hemorrhagic cystitis [15]. BKPyV infection is also ubiquitous in humans and detectable in the urine of healthy individuals [16]. Recently, it has been considered as one of the causative agents of nephropathy in renal transplant recipients. Thus, these two viruses are very important in the clinical setting.

In this study, we assessed the ability of the GP method to detect species of virus. Secondly, we compared the results of the GP method with the conventional identification method. Finally, in order to provide evidence that this method can be used in clinical cases, we conducted the GP method with urine samples from two individuals as a trial.

## 2 MATERIALS AND METHODS

### 2.1 DNA Samples

Whole genome plasmids of 13 JCPyV isolates [17] (Table **1**) and **3** BKPyV isolates [18] (Table **2**), using pUC-19 as a vector, were used in this study (1.0 ng/µL). For the clinical case study, we collected urine samples from a JCPyV positive individual and from a negative individual whose urinary infection was confirmed in another previous study using PCR amplification of the virus genome, and DNA was extracted using QIAmp DNA mini kits (QIAGEN, Tokyo, Japan) according to the manufacturer’s instructions. The final concentration of DNA was set as 1.0 ng/µL and used in the following experiments. This study was approved by the institutional review board (No. G-98-1).

### 2.2 GP Method

#### 2.2.1 Random PCR

The reaction mixture (25 μL) contained 17.5 pmol SP-1 primer (pfm12) (5′-agaacgcgcctg-3′) [19, 20], 0.16 mM each dNTP, 1× ExTaq Buffer, 0.5 U ExTaq polymerase (TaKaRa Bio, Inc., Shiga, Japan), and 1.0 ng of crude DNA. We checked other primers for random PCR in the preliminary experiment; however, SP-1 was the most successful primer for the random PCR.

PCR was performed using a PC-320 Thermal Cycler (ASTEC, Fukuoka, Japan). After denaturation at 94°C for 5 min, 30 cycles of 94°C for 30 s, 26°C for 1 min, and 47°C for 1 min were performed, followed by extension at 47°C for 5 min.

#### 2.2.2 Internal Standards [1]

M13 phage and pBR322 were used as the internal reference samples according to the GP method. Reference 1 (Ref1; M13 phage DNA) was approximately 200 bp, and reference 2 (Ref2; pBR322 DNA) was approximately 900 bp. Ref1 was PCR amplified using the primers MA1 (5′-tgctacgtctcttc-cgatgctgtctttcgc-3′) and MA2 (5′-ccttgaattctatcggtt-tatca-3′). The total reaction volume of 50 μL contained 1.0 μg M13 phage DNA (TaKaRa Bio, Inc.), 0.75 U ExTaq DNA polymerase, 200 μM dNTPs, 0.6 μM primers, and a PCR buffer supplied by the manufacturer. PCR amplification was performed for 30 cycles of 94°C for 30 s, 63°C for 1 min, and 72°C for 30 s, followed by a final step of 72°C for 5 min. Ref2 was PCR amplified using the primers Ref6F (5′-gccggcatcaccggcgcca-caggtgcggttg-3′) and Ref6R (5′-tagcgaggtgccgccggc-ttccattcaggtc-3′). The total reaction volume of 50 μL contained 0.25 μg pBR322 DNA (TaKaRa Bio, Inc.), 1.25 U ExTaq DNA polymerase, 200 μM dNTPs, 0.7 μM primers, and a PCR buffer supplied by the manufacturer. PCR amplification was performed for 25 cycles of 94°C for 15 s, 55°C for 30 s, and 72°C for 1 min, followed by a final step of 72°C for 30 s. 

#### 2.2.3 Temperature Gradient Gel Electrophoresis

The 0.3μL of two reference DNAs as internal reference standards described above and 1μL of 6X loading buffer were added to the 4.4μL amplified DNA samples. Then, the total 6μL of DNA sample was electrophoresed at a temperature gradient on a 6% polyacrylamide gel for 10 min at a constant voltage of 100 V [21, 22] using µTG (TAITEC, Saitama, Japan). The temperature gradient was 15–65°C. Then, the electrophoresed gel was stained in a 0.1 M NaCl solution containing 0.03% GelRed (Biotium, Inc., CA, USA) for 10 min and a photograph of the gel was taken under UV light using a LAS 4000 Mini (FUJIFILM, Tokyo, Japan). The sensitivity of the GP method was checked by visible amplified fragments using diluted plasmid DNA samples (X1, X10, X100, X1000, X10000, and X10000).

#### 2.4 Cluster Analysis

From the photograph of the electrophoresed gel, *spiddos*, which indicate melting points of ds DNA, were selected manually. The *spiddos *were adjusted using the *spiddos *of the reference internal standards, and Pattern Similarity Scores (PaSSs) were calculated using micro-temperature gradient gel electrophoresis analyzer software [23-28]. Below is the equation for PaSS calculation.

$\begin{array}{c} \mathrm{PaSS}=1-\frac{1}{n}\sum_{i=1}^{n}\frac{\left|{\vec{p}}_{i}-{{\vec{p}}_{i}}^{\prime }\right|}{\left|{\vec{p}}_{i}|+|{{\vec{p}}_{i}}^{\prime }\right|}\\ \left[\vec{p}=\left(\theta ,\mu \right)\right] \end{array}$

Difference of the coordinates of *spiddos *(*=(θ,μ)) *reflects the difference of the DNA sequence. The PaSS value represents the degree of similarity between genomes. The PaSS value ranges from 0 to 1.0; if the genomes match perfectly, then the PaSS value is 1.0. From the calculated PaSS values, all samples were cluster analyzed using the Ward method [29].

## 3 SEQUENCING AND PHYLOGENETIC ANALYSIS

### 3.1 Sequencing

A BigDye^®^ Terminator v3.1 Cycle Sequencing Kit (Life Technologies, Foster City, CA) was used in this study. All procedures were performed according to the manufacturer’s instructions. Briefly, 5 μL of reaction mixture contained 0.5 µL of sequencing primers P-1 (5′-cacaagcttttttgggacactaacaggagg-3′; nt 2107–2127) and P-2 (5′-gattctgcagcagaagactctggacatg-3′; nt 2762–2742 in the JCPyV [MadI] genome [GenBank accession no. J02226]) for JCPyV, or 327-1PST (5′-gcctgcagcaagtgc-caaaactactaat-3′; nt 1630–1649 in the BKPyV [Dunlop] genome [GenBank accession no. V01108; NCBI no. NC_0015^38^]) and 327-2HIN (5′-gcaagcttgcatgaagg-ttaagcatgc-3′; nt 1956–1937) for BKPyV [30], and 3 µL plasmid DNA. The cycle sequencing conditions were an initial step of 1 min at 96°C and 25 cycles of 10 s at 96°C, 5 s at 50°C, and 4 min at 60°C using a PC-320 Thermal Cycler. A 310 Genetic Analyzer (Life Tech-nologies, city, CA) was used to determine the 610 bp IG region of JCPyV or 287 bp typing region of BKPyV. By sequencing these typing regions of both viruses, we confirmed the virus isolates. After confirmation, we downloaded the whole genome data of all isolates from GenBank (http://www.ncbi.nlm.nih.gov/) at the National Center for Biotechnology Information.

### 3.2 Phylogenetic Analysis

Using the downloaded whole genome sequences of all isolates, phylogenetic analysis was performed using the neighbor-joining (NJ) method with maximum substitution. Molecular Evolution Genetic Analysis ver.4 [31] was used for the analysis.

### 4 STATISTICAL ANALYSIS

Statistical analysis was performed using the Student’s *t *test, with the significance set at the 1% level

## 5 RESULTS

**Fig. (2):**
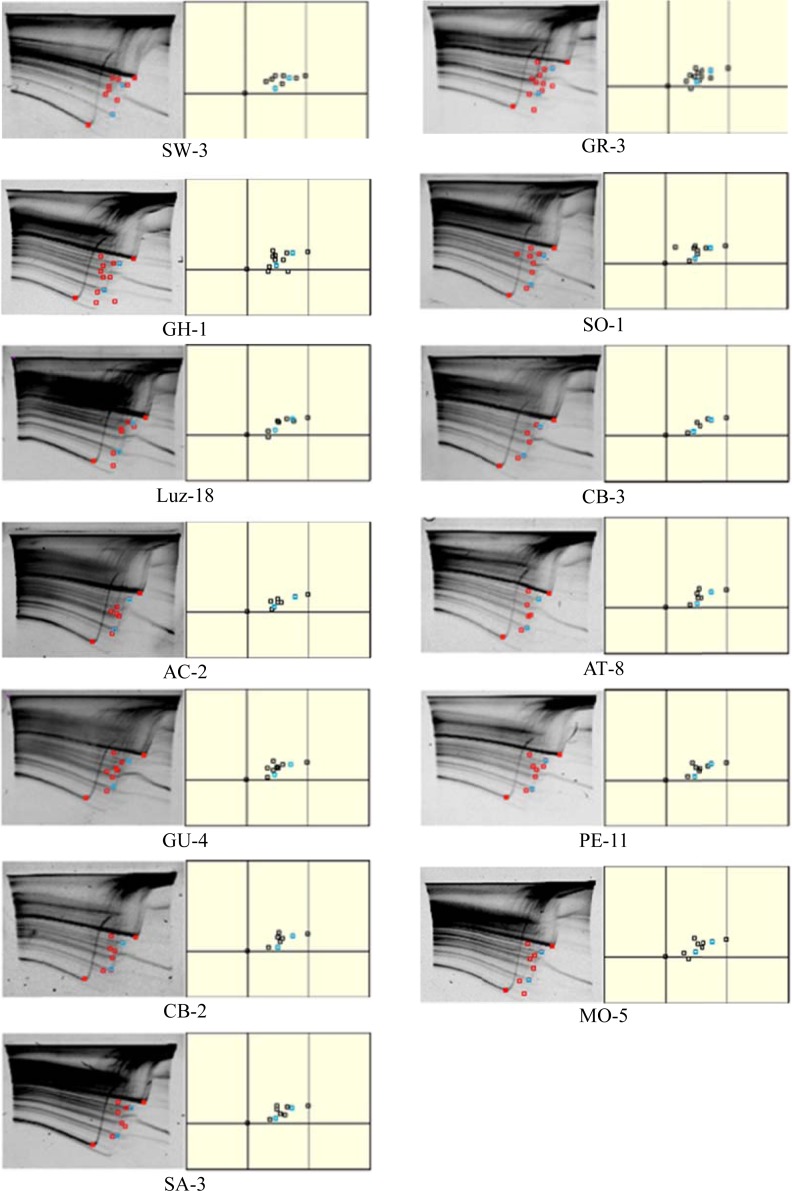
UV photographs of temperature gradient gels of randomly amplified JCPyV samples stained with GelRed (left) and standardized *spiddos* (right). Obtained *spiddos* are shown by open red boxes and the reference *spiddos* are shown by closed red boxes. The common *spiddos* found in the same virus species are shown by blue boxes.

**Fig. (3):**
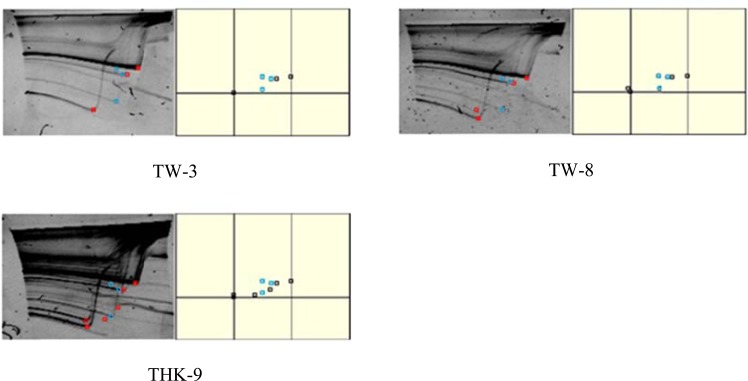
UV photographs of temperature gradient gels of randomly amplified BKPyV samples stained with GelRed (left) and standardized *spiddos* (right). Obtained *spiddos* are shown by open red boxes and the reference *spiddos* are shown by closed red boxes. The common *spiddos* found in the same virus species are shown by blue boxes.

For the GP method, the average number of *spiddos *obtained was 8.23 for JCPyV and 5.0 for BKPyV (Table **3**). The amplified DNA fragments were observed in the samples diluted up to X10000. The number of common *spiddos *in the same virus species was 2 for JCPyV (Fig. **2**) and 3 for BKPyV (Fig. **3**). The PaSSs were 0.9757–0.9895 among JCPyV isolates (mean ± standard deviation [SD]: 0.9836 ± 0.0026) and 0.9792–0.9853 among BKPyV isolates (mean ± SD: 0.9828 ± 0.0032) (Table **4**). Comparing the BKPyV isolates to the JCPyV isolates, the PaSS value among BKPYV isolates was 0.9632-0.9802 (0.9710 ± 0.0039) (Table **4**). There was a statistically significant difference between the PaSS values among these two virus species (P<0.01). When the GP method was replicated, the PaSS values in the same virus strain were 0.9768–0.9893 (mean ± SD: 0.9841 ± 0.0036).

The results of cluster analysis after the GP method are shown in Fig. (**4**). The JCPyV and BKPyV clusters were apparently independent. The JCPyV strains formed two major clusters. One of the clusters contained the SW-3, GR-3, GH-1, and CB-2 strains, while the other cluster contained the remaining strains. 

**Fig. (5):**
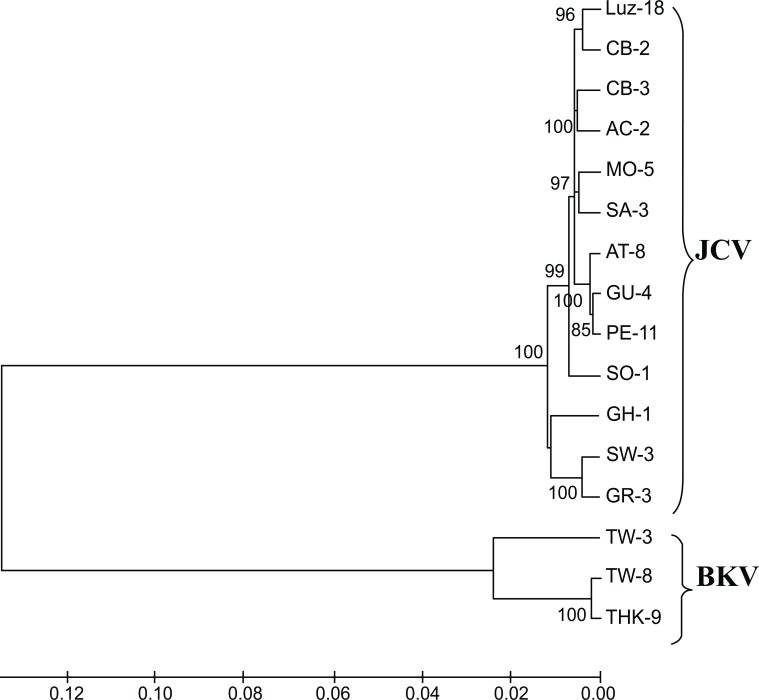
Phylogenetic analysis of JCPyV and BKPyV whole genomes. NJ phylogenetic tree showing the relationship between the JCPyV and BKPyV isolates. The numbers at the nodes indicate the bootstrap confidence levels (percent) obtained with 1,000 replicates [40].

The results of NJ phylogenetic analysis using whole genome sequences are shown in Fig. **(5)**. The SW-3, GR-3, and GH-1 strains formed one cluster. As all JCPyV strains classified by the conventional method were not the same as that classified by the GP method, we were unable to distinguish the JCPyV and BKPyV genotypes perfectly using the latter method. However we were able at least to distinguish between the two virus species.

**Fig. (6):**
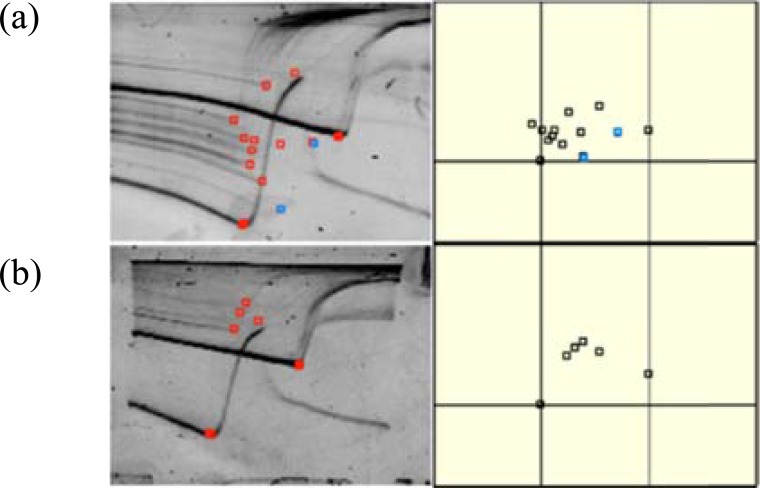
UV photographs of temperature gradient gels and standardized *spiddos* of clinical samples. **(a)** UV photographs of temperature gradient gels of randomly amplified JCPyV positive urine sample stained with GelRed (left) and standardized *spiddos* (right). Obtained *spiddos* are shown by open red boxes and the reference *spiddos* are shown by closed red boxes. The common *spiddos* found in the same virus species are shown by blue boxes. **(b)** UV photographs of temperature gradient gels of randomly amplified JCPyV negative urine sample stained with GelRed (left) and standardized *spiddos *(right). Obtained *spiddos* are shown by open red boxes and the reference *spiddos* are shown by closed red boxes.

Finally, we analyzed clinical samples. Fig. **(6a)** shows the results of the GP method using the JCPyV positive urine sample. JCPyV specific *spiddos *were obtained in this sample. There were also numerous *spiddos *obtained from this sample. Those obtained *spiddos *were different from those of the plasmid DNA samples. Sequencing analysis was also performed on this sample, and this JCPyV genotype was CY. Fig. **(6b)** shows the results from the GP method using the JCPyV negative urine sample. There were no JCPyV specific *spiddos *obtained in this sample. 

## 6 DISCUSSION

Though the homology between JCPyV and BKPyV is about 70%, the number of the *spiddos* were much higher in the JCPyV. This is because the random primer attaches to and amplifies different DNA sequences. The homology between JCPyV strains is about 99%; however, several different *spiddos *are obtained. It also is considered that amplified DNA fragments might include vector DNA as well as virus DNA. However, the virus specific *spiddos *show that among those amplified fragments, virus DNA were surely amplified, and the number of the common *spiddos *is 2 or 3. Though these numbers are relatively small, if we consider the small size of virus genome, it is reasonable. Small differences, such as between the isolates in the same virus species, are calculated by the PaSS score. So the number of the *spiddos *is not used for the determination of virus species. The number of the *spiddos *is important for the calculation of PaSS. If the number of the *spiddos *is rich, we can get more reliable PaSS results. Because of the random PCR system, we cannot avoid the amplification of other contaminated DNA. Therefore specific *spiddos *are the most important for the diagnosis of those viruses.

The results of cluster analysis obtained using the GP method did not agree completely with those obtained using phylogenetic analysis. Although we could discriminate between virus species, we could not discriminate virus genotype with the GP method. We consider one of the main reasons for this to be that cluster analysis does not include molecular evolution aspects; it only classifies a similar *spiddo *pattern that represents a genome sequence. In addition, as around 70% of the genome sequence of BKPyV is the same as that of JCPyV, they can be distinguished using the GP method. However, because around only 1% of the genome sequence is different among the JCPyV or BKPyV isolates, it might be difficult to distinguish such small differences using the GP method. These small differences are very important for us to analyze population history not only in the studies using JCPyV and BKPyV [17, 32-37] but also in other studies [38, 39]. In the current GP method, because the *spiddos *are selected manually, the researcher’s technique may affect the reproducibility of the results. In our previous study, when one highly skilled researcher performed the GP method 5 times using the same sample, the PaSS value was 0.9905 ± 0.0024. If the GP method had complete reproducibility, the PaSS value should have been 1.0. As a result, the difference of the PaSS value in the GP procedure was around 1% [2]. Differences of electrophoresis are standardized with the use of internal standards, and these differences do not affect the PaSS value. In this study, we performed the GP method twice using the same samples, and the difference in the PaSS value was 0.9941 ± 0.0036. It is considered that this difference was also the result of the manual procedure. To improve reproducibility, it may be necessary not only to increase the number of trials and improve researchers’ technical skills, but also to perform automated selection of *spiddos *using special software. If the *spiddos *are selected automatically, we will always be able to obtain the same *spiddos*; the PaSS values will be 1.0. Finally, the *spiddos *used in the calculation of PaSS might include the amplified fragment of the vector DNA. That dilute the difference among the each viral strains. Because of the small genome size of those viruses, the number of the specific *spiddos *is low. Therefore, we consider it might much more reliable to use all obtained *spiddos *for the calculation. 

In this study, the whole procedure required around 6 hours using the GP method and around 36 hours for the conventional method. Especially, checking the obtained sequence in the electropherograms generated by the sequencer requires a considerable amount of time. The GP method does not require such time-consuming procedures or expensive reagents and machines. 

The sensitivity of the GP method was also very high. However, the detection from plasmid DNA samples may different from the detection from clinical urine samples. We will try to examine the sensitivity using the clinical materials.

Some of the *spiddos *were present only in JCPyV or BKPyV. These virus-specific *spiddos *represent virus-specific sequences. Therefore, if many kinds of virus genome are analyzed in advance with the GP method, it may be possible to identify virus species by detecting virus-specific *spiddos*.

In our trials using clinical samples, we could detect many *spiddos *(Fig. **6**). In the JCPyV positive urine sample (Fig. **6a**), there were also numerous *spiddos *obtained from this sample. These *spiddos *might have resulted from the amplification of human DNA contained in the urine. The number of human cells in the urine varies with the individual, and obtained *spiddos *were different from those of plasmid DNA samples. That might be due to the fact that most of the materials for amplification were used to amplify the human DNA. However, we could obtain the virus specific *spiddos*. In the JCPyV negative urine sample (Fig. **6b**), there were no JCPyV specific *spiddos *obtained. Neither could we obtain many *spiddos *in this sample, indicating that the urine contain little DNA of human or of microorganisms. The amount of human DNA in the urine may be also affected by the urinary virus infection. With this result, we consider that we may be able to utilize the GP method to detect polyomavirus in urine samples. Of course, for this purpose it is necessary to validate this method with a large number of cases.

## CONCLUSION

In this study we showed that the GP method can be used to discriminate among different human polyoma-virus species. In addition, it may be used for other virus species. The GP method is a very simple and inexpensive tool to analyze virus genomes. It does not require virus-specific primers, so although it cannot distinguish virus genotypes, it may be used for virus screening. We demonstrated the possibility to utilize this method to detect urinary viruses using test clinical test cases. We plan to analyze various viruses in clinical samples in the near future.

## Figures and Tables

**Fig. (4):**
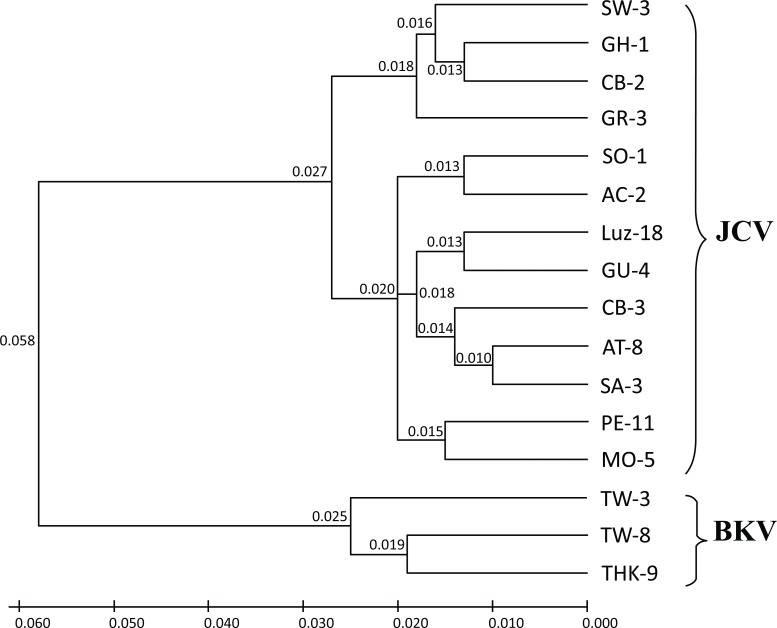
Cluster analysis as a result of the GP method using JCPyV and BKPyV whole genomes. The horizontal axis and numbers at the nodes show the genome distance. The JCPyV and BKPyV isolates are clearly distinguished.

**Table 1:** JCV isolates used in this study.

Isolate	Genotype	Accession no.	Reference
SW-3	Eu-a	AB048575	[31]
GR-3	Eu-b	AB048563	[31]
GH-1	Af1	AB038252	[32]
SO-1	Af2-a	AB127012	[33]
Luz-18	SC	AB113130	[34]
CB-3	CY-a	AB048560	[31]
AC-2	CY-b	AB212953	
AT-8	MY-a	AB048577	[31]
GU-4	MY-e	AB081014	[35]
PE-11	MY-f	AB081024	[35]
CB-2	B1-a	AB048550	[31]
MO-5	B1-b1	AB048552	[31]
SA-3	B1-d	AB048555	[31]

**Table 2:** BKPyV isolates used in this study.

Isolate	Genotype	Accession no.	Reference
TW-3	IV	AB211391	[36]
TW-8	Ic	AB211385	[36]
THK-9	Ic	AB211379	[36]

**Table 3:** Number of *spiddos* obtained with temperature gradient gel electrophoresis.

JCPyV strains		
Isolate	Genotype	No. of *spiddos*
SW-3	Eu-a	8
GR-3	Eu-b	12
GH-1	Af1	11
SO-1	Af2-a	8
Luz-18	SC	8
CB-3	CY-a	6
AC-2	CY-b	7
AT-8	MY-a	7
GU-4	MY-e	9
PE-11	MY-f	8
CB-2	B1-a	7
MO-5	B1-b1	8
SA-3	B1-d	8
BKPyV strains		
TW-3	IV	4
TW-8	Ic	5
THK-9	Ic	6

**Table 4:** PaSSs generated in this study.